# Health Tract No. 337

**Published:** 1868-07

**Authors:** 


### HEALTH TRACT No. 337.

### ARCHITECTS

Are men who furnish plans for buildings, sometimes including the supervision of their erection, to see that the structure is made according to the plan, with good materials and in a workmanlike manner ; they ought to be men of superior education, especialy in mathematics, chemistry and natural philosophy, understanding thoroughly the laws of heat, air, vegetable growths, mineralogy, hygrometry, acoustics, ventilation, drainage, &c. It is doubtful whether there lives on the globe, one man who knows how to build a house, public or private, fit for the purposes designed. Whoever saw a church that did not leak, unless it had an old fashoned hip roof? who was ever in a church which would comfortably seat five hundred persons, who could hear the minister without an effort? where is the private dwelling on the face of the globe which is agreeably and healthfully warmed in winter time? What millions of money are lost every year by fires originating in "defective flues"? One may go through whole blocks of "brown stones" worth thirty thousand each and find in every one of them such an arrangement of flues and registers, that no heat is given to the first, second or third floor, unless the flue on the fourth floor is closed; none on the second, unless those on the third and fourth are closed, the uppermost flue open, getting all the heat; is it possible for any man, not a born idot, to devise a more absurd system of heating a house than that where only one front room in a building can be heated at a time. The a. b. c. of architecture ought to have indicated that each room of a private dwelling should be independent of all others for its warmth. A New York City architect has dug a cellar in sight of us, having a "hard pan" floor one foot below the drainage of the city! In the elegant building of the New York Times, there is said to be not one square foot of ventilation, either exhaust or supply, nor in Bonner's new Ledger Building, nor in Harper's mammoth establishment. That magnificent structure, the New York Herald building, not only has no ventilation but the arrangements are such that all the escape gas and heat from the boilers and smell from the printing rooms in the basement, are not allowed to escape in winter but are distrbuted through the building. The Ledger building of Philadelphia is about the same. The Sun building, formerly "Old Tammany" is ditto. The new Park Bank is reported to have no ventilation, the parties declaring it to be as tight as the "States Prison." The new building for the " New York Life Insurance Co." could not be ventilated as it was all glass and iron and there was no room whatever for ventilating flues." Leeds says, " to the Cooper Institute reading room of New York there is no provision for a regular supply of fresh air, not one foot, not one inch, neither are there any regular flues for the removal of foul air. He reports also that in "visiting the New York Public Schools, registers were in all the rooms, top and bottom, beautiful green and blue tassels and cords to adjust them, but on going to the top of the building the flues were covered air tight with solid stone coping."

Have we an architect among us?

